# Clinical and cost-effectiveness of physiotherapy interventions following total knee replacement: a systematic review and meta-analysis

**DOI:** 10.1007/s00402-021-03784-5

**Published:** 2021-02-07

**Authors:** F. Fatoye, G. Yeowell, J. M. Wright, T. Gebrye

**Affiliations:** Department of Health Professions, Faculty of Health, Psychology, and Social Care, Manchester Metropolitan University, Brooks Building, 53 Bonsall Street, Manchester, M15 6GX UK

**Keywords:** Cost-effectiveness analysis, Total knee replacement, Physiotherapy, Systematic review

## Abstract

**Purpose:**

Osteoarthritis is the single most common cause of pain and disability in older adults. This review addresses the question of the clinical effectiveness and cost-effectiveness of physiotherapy interventions following total knee replacement (TKR).

**Methods:**

A systematic review was conducted according to the Preferred Reporting Items for Systematic Reviews and Meta-Analyses. MEDLINE, CINAHL, AMED, DARE, HTA and NHS EED databases were searched from inception to 02 May 2020. Search terms related to the clinical and cost-effectiveness of physiotherapy interventions were used. Studies meeting the inclusion criteria were identified and key data were extracted. Random effect meta-analysis was conducted for pain, physical function and range of motion (ROM).

**Results:**

In total, 1467 studies were identified. Of these, 26 studies were included; methodological quality of most studies was adequate. Physiotherapy interventions were more effective than control for function, SMD − 0.166 [95% Confidence Interval (CI) − 0.420 to 0.088.] and ROM, SMD − 0.219 [95% CI − 0.465 to 0.028] for a follow-up of 2 or 3 months. Patients in the intervention group showed improvement in pain at 12–13 weeks, SMD − 0.175 [95% CI − 0.416 to 0.067]. No evidence on the pooled estimate of cost-effectiveness of physiotherapy interventions was found.

**Conclusions:**

This is the first systematic review and meta-analysis that has examined the clinical and cost-effectiveness of physiotherapy interventions following TKR. The findings of this review suggest that physiotherapy interventions were effective for improving physical function, ROM and pain in a short-term follow-up following TKR. Insufficient evidence exists to establish the benefit of physiotherapy in the long term for patient with TKR. Further study should examine the long-term effectiveness and cost-effectiveness of physiotherapy interventions.

## Introduction

Osteoarthritis (OA) is a slowly progressive musculoskeletal disorder where the knee is the most common joint affected [1]. It is a major public health problem due to its prevalence, physical disability and high economic burden. The prevalence of knee OA was highest in high-income Asian Pacific regions such as Japan and South Korea [2]. Moreover, the prevalence of knee OA in people aged ≥ 45 years was projected to increase from 13.8% in 2012 to 15.7% in 2032 [3]. OA of the knee tends to be more prevalent in women [4]. Additional factors that contribute to the development of OA include knee injury, being overweight and obesity, old age, muscle weakness, repetitive use of joints, and bone density. The global age-standardised point prevalence and annual incidence rate of OA in 2017 were 3754.2 and 181.2 per 100,000, respectively [5]. This is an increase of 9.3% for the point prevalence and 8.2% for the annual incidence rate from 1990. It is also highly likely to rise due to an increasing aging population and obesity [6].

It has been noted that OA is a leading indication for the use of pharmacological treatments [7]. The findings of a systematic review [8] suggested that the efficacy of all pharmacological treatments of knee OA significantly outperformed oral placebo for pain and function. However, when pharmacological and non-pharmacological treatments fail, surgery is recommended for patients with knee OA [9]. Total knee replacement (TKR) is a surgical procedure aimed at restoring function and resolving pain of knee OA [10]. In the majority of western healthcare system such as the United Kingdom between 150 and 250 per 100,000 of the population undergone TKR annually [11]. It is also estimated that a total of 3.48 million TKR per year will be performed by the year 2030 in the United States; however, approximately 1 out of 5 people that undergo TKR remain unsatisfied even with new technological advances such as knee kinematics [12].

Non-pharmacological interventions such as rehabilitation are an integral part of the overall recovery process following TKR as well as essential to improve clinical outcomes [13]. A systematic review and meta-analysis of physiotherapy exercise concluded that physiotherapy exercises were beneficial in the short term and provided small to moderate benefit for function, range of motion and quality of life (QoL) 3–4 months following TKR [13]. On the other hand, a non-significant difference was reported for physical function and knee range of motion between outpatient physiotherapy and home-based exercise regimes in patients following TKR [15]. In 2002, costs of arthroscopic surgery for OA and indirect costs from OA in the United Kingdom were estimated at £1.34 million and £3.2 billion, respectively [16]. Furthermore, older patients with OA in the United States of America spend annually on average $8601, $2941, and $4603 for direct medical, drug and indirect work loss costs, respectively [7]. Given the range of physiotherapy interventions recommended for patients following TKR, it is important to consider their economic costs as well as clinical effectiveness in allocating healthcare resources [16]. The selection of a particular intervention depends not only on its clinical decisions but also its value for money (cost-effectiveness). By comparing the costs and health effects of an intervention, cost-effectiveness analysis is important to investigate the extent to which it can be regarded as providing value for money [18].

Although several systematic reviews have been published [14, 15, 19], they do not include recently published evidence nor consider the cost-effectiveness of physiotherapy interventions. The aim of this systematic review was, therefore, to: (1) update and synthesise the clinical effectiveness of physiotherapy interventions following TKR (2) summarize the cost-effectiveness of physiotherapy interventions following TKR.

## Methods

**Fig. 1:**
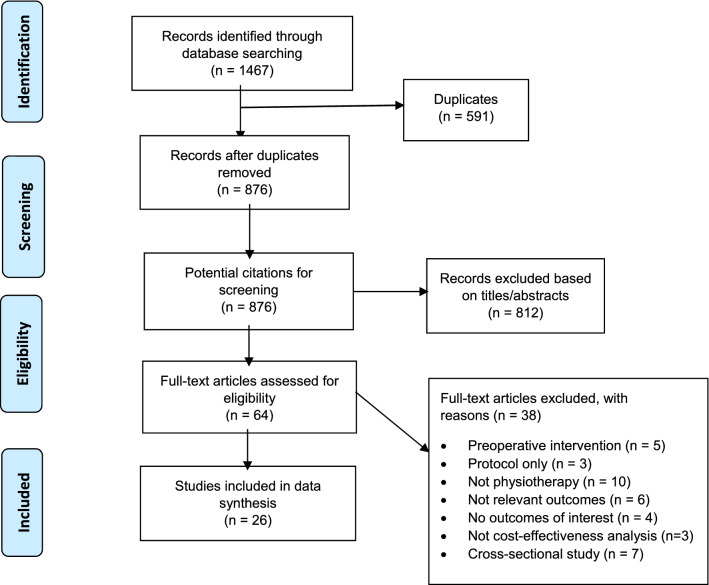
Systematic review flow diagram

This systematic review used the Preferred Reporting Items for Systematic Reviews and Meta-Analysis (PRISMA) (Fig. 1), a technique that addresses the eligibility, data sources, selection of studies, data extraction and data analysis as a reporting guideline [20]. This review was registered on PROSPERO, with registration number, CRD: CRD42018096524.

### Information sources

We searched MEDLINE, CINAHL, AMED, DARE, HTA and NHS EED databases from inception to 02 May 2020. All searches were limited to humans, English language, publication data and abstract available. We also hand searched from the references of key studies included in the review. Search results were screened for relevance according to the eligibility criteria outlined in Table 1.

**Table 1:** The eligibility criteria

Inclusion	Exclusion
Adults following TKR	Systematic reviews
Physiotherapy interventions	Conferences
Standard care and no intervention were used as a comparator	Abstracts
Pain, function, health-related quality of life (QoL), range of motion for clinical effectiveness and cost per quality adjusted life year (QALY) for cost-effectiveness	Case reports, and dissertations
Randomised controlled trail for clinical effectiveness studies	

The search and screening process was done by a team of systematic reviewers (FF, GY, JMW and TG) led by FF. We obtained full text for studies that seemed potentially relevant based on the title and abstracts. Two reviewers (FF and TG) independently assessed the full-text articles and selected studies that met the inclusion criteria. Reference lists of relevant review articles that met the inclusion criteria were also searched for articles. Any discrepancies between the reviewers were resolved by discussion with the other authors (GY and JMW).

### Study selection and quality assessment

Two researchers (TG and FF) independently undertook the study quality assessment of the articles. Titles and abstracts that did not provide enough information regarding the eligibility criteria were considered for full-text evaluation. Full-text articles were printed for further reading and to assess if they met the inclusion criteria. Any disagreements with regard to the study selection were resolved by discussion with the other authors (GY and JMW). The methodological quality of the included clinical effectiveness studies was assessed based on a tool recommended by Maher and colleagues [21]. This tool is a Physiotherapy Evidence Database (PEDro) scale designed for rating methodological quality of randomised controlled trials. The following cut-off points were used to determine the level of quality of the studies: 9–10, excellent; 6–8, good; 4–5, fair; and < 4, poor. Studies with a total score of at least six points were considered to be of adequate quality [21]. To assess the quality of reporting of the included cost-effectiveness studies, we completed the Consolidated Health Economic Evaluation Reporting Standards (CHEERS) statement [22]. The CHEERS statement contains a 24-item checklist. A total score of 1 was assigned if they fulfilled the requirement of reporting for that Item completely, 0 for not reporting and 0.5 for partial reporting. The maximum score for an article that reported completely all information was 24. Studies with a score ≥ 20 out of total 24 were considered good quality whereas those less than 20 poor quality [22].

### Data extraction and analysis

Two researchers (FF and TG) were involved in extracting the data from the included studies. For each of the included studies, the following data were extracted: author and date of the study, the location/country, type of patients, and the number of participants involved in the study were extracted. The mean age, participants receiving the interventions and the control arms, length of follow-up, and the perspectives of the economic evaluation were extracted from each included study. Furthermore, data regarding results of the studies including pain, function, range of motion, health-related QoL were extracted for the clinical effectiveness studies. For the cost-effectiveness studies, data relating to cost per QALY or disability-adjusted life year (DALY) were extracted.

A descriptive synthesis and meta-analysis of the extracted data are presented. Meta-analysis was conducted using the Forest plot. Forest plot enables to illustrate results of selected studies graphically in a meaningful way. For the included clinical effectiveness and cost-effectiveness studies, relevant data including mean, standard deviation, and sample size wherever available were collected for quantitative synthesis. We used the Cohen d as the effect size index. Effect size of each study was entered in to the Comprehensive Meta-analysis Software and were calculated for the intervention group relative to the comparison group. A random effect model was used to account for heterogeneity both within- and between studies. The type of interventions, the duration of the intervention and the source of the outcome measures were the main factors for estimating the pooled effect size of the included studies.

## Results

The steps followed to select the studies for this review are presented in Fig. 1. The literature search strategy yielded 1467 records. Of these, after adjusting for duplicates 876 abstracts remained for consideration. After the titles and abstracts of the studies were screened, 64 full-text copies were obtained for further reading. Twenty-three studies met the inclusion criteria for the clinical effectiveness aspect of the review and 3 studies fulfilled the inclusion criteria for the cost-effectiveness. The methodological quality of the clinical effectiveness studies was assessed as adequate quality (> 6 point score) (Table 2). As indicated in Table 4, three of the included cost-effectiveness studies were of good quality, with scores ranging from 21 to 23.

**Table 2:** Methodological quality of the included clinical effectiveness studies (PEDro scale)

Study	PEDro scale											
	1	2	3	4	5	6	7	8	9	10	11	Total
Codine et al. [23]	1	1	0	1	0	0	1	1	1	1	1	8
Liu et al. [24]	1	0	0	1	0	0	1	1	0	1	1	6
Haas et al. [25]	1	0	0	1	0	0	1	0	1	1	1	6
Mitchell et al. [26]	1	0	1	0	0	1	1	1	0	1	1	9
Herbold et al. [27]	1	0	0	1	0	0	1	1	0	1	1	6
Hasubhai et al. [28]	1	1	0	1	0	0	1	1	0	1	1	7
Kauppila et al. [29]	1	0	0	1	0	0	1	1	0	1	1	6
Kramer et al. [30]	1	1	0	1	0	0	1	1	0	1	1	7
Artz et al. [31]	1	1	0	1	0	0	1	1	0	1	1	7
Frost et al. [32]	1	1	1	1	1	1	1	1	0	1	1	10
Mockford and Beverland [33]	1	1	0	1	1	1	1	1	0	1	1	9
Rajan et al. [34]	1	1	0	1	0	0	1	1	0	1	1	7
Bruun-Olsen et al. [35]	1	1	1	1	1	1	1	1	1	1	1	11
Evgeniadis et al. [36]	1	1	1	1	1	1	0	1	0	1	1	8
Madsen et al. [37]	1	1	0	1	0	0	1	1	0	1	1	7
Minns Lowe et al. [38]	1	1	1	1	1	1	1	1	0	1	1	10
Moffet et al. [39]	1	1	0	1	1	1	1	1	0	1	1	9
Monticone et al. [40]	1	1	0	1	1	1	1	1	0	1	1	9
Wang et al. [41]	1	0	0	1	0	0	1	1	0	1	1	6
Lenssen et al. [42]	1	1	1	1	1	1	1	1	1	1	1	11
Donec and Krisciunas [43]	1	1	0	1	1	1	1	1	1	1	1	10
Denis et al. [44]	1	1	1	1	1	1	1	1	1	1	1	11
Avramidis et al. [45]	1	1	0	1	0	0	1	1	0	1	1	7

Note: 0 indicates no; 1 indicates yes

### Characteristics of the studies

The studies included in this review evaluated the clinical effectiveness (*n* = 23) and cost-effectiveness (*n* = 3) of physiotherapy interventions using 2642 individuals following total knee replacement (Tables 3 and 4). The mean age of the patients that received physiotherapy interventions and control ranged from 64.1 to 74.6 and 65 to 75, respectively. The duration of follow-up ranged from 1 week and 12 months. The included studies were conducted in UK (*n* = 8), USA (*n* = 2), Finland (*n* = 2), Canada (*n* = 3), Slovakia, Lithuania, India, Norway, Greece, Denmark, Italy, Netherlands, France, China, and Australia.

**Table 3:** Clinical effectiveness study characteristics of trials evaluated in systematic review

Author, year, location	Patients				Intervention time	Results
	No	Type	Mean (SD) age	% Female		
Codine et al. [23], France	60 (In = 30; Cot = 30)	Patients who underwent unilateral TKR	Int = 74.6 (13) Cot = 71.14 (15)	Int = 53Cot = 70	4 weeks	Int, Range of Motion (ROM flexion) = 102.32 (7.75); extension = − 1.25 (2.2) Cot, Range of Motion (ROM flexion) = 104.64 (7.8); extension = − 2.67 (3.72); both measured in degrees and they are non-significant
Liu et al. [24], China	86 (Int = 43, Cot = 43)	Patients who underwent TKR	Int = 72 (7.4) Cot = 73.3 (6.9)	N/A	13 weeks	WOMAC Int; pain = −10.1 (− 14.1, − 7.9); *p* = 0.25; function = − 30.9 (− 39.5 to 22.4) *p* = 0.16 Cot pain = − 8.3 (− 11.6 to 6.7); function = − 26.6 (− 32.7 to 19.8) KOOS Int; pain = − 56.4 (− 69.8, 42.1); function = − 53.8 (− 61.7 to − 44.5); quality of life = − 37.9 (− 48.2, − 29.3) Cot; pain = − 47.2 (− 62.9 to − 35.5); function = − 45.3 (− 53.0 to − 38.7); quality of life = − 28.7 (− 36.9 to − 20.3)
Haas et al. [25], Australia	217 (Int = 130; Cot = 87)	Patients who underwent a lower limb knee joint replacement	Int = 67.77 (10.62) Cot = 68.58	Int = 58Cot = 62	12 months	Acute hospital length of stay, mean difference (MD) = 3.151 (1.039–9.555) improved mobility, MD = 4.301 (1.5–7.101)

Author, year, location	Patients				Intervention time	Results
	No	Type	Mean (SD) age	% female		
Mitchell et al. [26], UK	114 (Int = 57; Cot = 57)	Patients who underwent TKR	Int = 70.0 (7.2) Cot = 70.6 (8.2)	Int = 63.2 Cot = 52.6	12 weeks	Health related QoL, MD = 0.002 (− 0.034 to 0.039) Western Ontario McMaster Osteoarthritis Index (WOMAC), functional daily living, MD = − 1.0 (− 5.9 to 3.8); *p* = 0.677 pain, MD = − 0.5 (− 2.0 to 1.0); *p* = 0.53 and stiffness, MD = − 0.2 (− 0.9 to 0.4), *p* = 0.496
Herbold et al. [27], USA	122 (Int = 61; Cot = 61)	Patient with unilateral TKR	Int = 69.72 Cot = 70.02	Int = 73.8 Cot = 72.1	1 week	Int, discharge active knee flexion ROM = 81.56, *p* = 0.084; flexion gain = 15.34, *p* = 0.086 Cot, discharge active knee flexion ROM = 84.20, *p* = 0.084; flexion gain = 17.98, *p* = 0.086
Kauppila et al. [29], Slovakia	40 (Int = 20, Cot = 20)	Patients who underwent unilateral TKR	Int = 67.9 (3.5) Cot = 68.2 (3.7)	100	3 months	Int, (3 months) VAS (quality of life) = 3.0 (0.8); (6 months) VAS = 1.8 (1.3) Cot, (3 months) VAS = 4.3 (0.7); (6 months) VAS = 2.6 (0.9)
Donec and Krisciunas [43], Lithuania	90 (Int = 40; Con = 49)	Patient with unilateral TKR	Int = 66.44 (8.10) Cont = 66.58 (8.19)	Int = 87.5%, Cot = 83.7%	4 weeks	Int: Knee pain intensity, (4 weeks) 2.2 (1.2) *p* < 0.001; knee flexion and extension (4 weeks) 100.6 (9.6°), 176.3 (5.2°) *p* < 0.001 Cot, pain intensity 2.9 (1.2) *p* < 0.006, knee flexion and extension 97.1 (12.3°) and 173.1 (6.2°) *p* < 0.001

Author, year, location	Patients				Intervention time	Results
	No	Type	Mean (SD) age	% female		
Hasubhai et al. [28], India	34 (Int = 17; Cot = 17)	Patients following TKR	N/A	Int = 76 Cot = 88.2	8 weeks	Int, pain = 1.65 (0.93) *p* = 0.37; knee flexion ROM = 4 (0) *p* = 1.00 and GROC = 6.41(0.62); Cot, pain = 1.88 (0.92); *p* = 0.00, knee flexion ROM = 4 (0), and GROC = 4.88 (0.70)
Artz et al. [31], UK	46 (Int = 23, Cot = 23)	Patients undergoing TKR	68.6 (51–82)	35	3 months 6 months	Int, 3 months, pain (VAS) = 3.5 (3.1); KOOS (function) = 81.2 (15.9); KOOS (pain) = 74.1 (19.9) 6 months, pain (VAS) = 2.9 (3.4); KOOS activity of daily living = 79.6 (23.4); KOOS (pain) = 78.6 (25.9) Cot, 3 months, pain (VAS) = 3.6 (2.2), KOOS (function) = 76.1 (18.5); KOOS (pain) = 69.1 (19.5) 6 months, pain (VAS) = 3.9 (3.6); KOOS activity of daily living = 73.5 (26.4); KOOS (pain) = 70.9 (27.1)
Frost et al. [32], UK	27 (Int = 16, Cot = 11)	Patients undergoing TKR	Int = 71.5 (5.4) Cot = 71.1(5.6)	Int = 11/23 Cot = 12/24	12 months	Int, 12 months–pain (OKS) = 2.5 (1.06); ROM = − 102 (9.3); 6 months–pain (OKS) = 2.1 (1.08); ROM = − 102 (9.3) Cot, 12 months–pain (OKS) = 1.5 (0.93); ROM = − 102 (16.2) 6 months, pain (OKS) = 2 (1.13); ROM = − 100 (15.3)
Kramer et al. [30], Canada	127 (Int = 62, Cot = 65)	Patients following TKR	68 (8)	N/A	12 months	Function (WOMAC scores) MD = − 0.18 (− 0.53 to 0.18). Walking (speed) MD = − 0.23 (− 0.60 to 0.14); ROM, MD = − 2.00 (− 6.58 to 2.60)

Author, year, location	Patients				Intervention time	Results
	No	Type	Mean (SD) age	% female		
Mockford and Beverland [33], UK	143 (Int = 71; Cot = 72)	Patients undergoing primary TKR	N/A	N/A	12 months	Int, 3 months, function = 26.3 (8.3); ROM = − 102 (10.8) 12 months, function = 25.9 (9.3); ROM = − 107.9 (11.4) Cot, 3 months, function = 29.5 (9); ROM = − 98 (11.6) 12 months, function = 71 (24.7); ROM = − 106.6 (13)
Rajan et al. [34], UK	116 (Int = 56, Cot = 60)	Patients undergoing TKR	Int = 69 (9.3) Cot = 68 (10)	Int = 20/36 Cot = 23/37	12 months	Int, ROM (3 months) = − 98 (8.1); ROM (12 months) = − 95 (8.5) Cot, ROM (3 months) = − 96 (7.1); ROM (12 months) = − 92 (8.2)
Bruun-Olsen et al. [35], 2013, Norway	57 (Int = 29; Cot = 28)	Patients undergoing primary TKR	69 (± 9)	56.1	9 months	Int, ROM (degrees) = 118 (7); KOOS (pain) = 82 (21); KOOS (function) = 81 (18); KOOS (quality of life) = 72 (24) Cot, ROM = 114 (17); pain (KOOS) = 74 (23); function (KOOS) = 75 (21)
Evgeniadis et al. [36], Greece	35 (Int = 15; Cot = 20)	Patients undergoing TKR	68.76 ± 5.64 + E21:G21	56.3	4 months	Int, function = 0.14 (0.39); ROM = − 98.42 (11.3) Cot, function = 0.38 (0.56); ROM = − 80.42 (10.2)
Madsen et al. [37], Denmark	80 (Int = 40; Cot = 40)	Patients undergoing primary TKR	66.6	41	6 months	Int, physical function (3/4 months = − 7.9 (5.9)); and (6 months—0.5 (7.5)) Cot, physical function (3/4 months = − 8.4 (6.7)); and (6 months − 9.7 (8.5))

Author, year, location	Patients				Intervention time	Results
	No	Type	Mean (SD) age	% Female		
Minns Lowe et al. [38], UK	107 (Int = 56; Cot = 51)	Primary TKR	69.2	58	12 months	Int, physical function (3/4, 6, and 12 months) = 70.57 (16.646); 76.98 (17.364); 81.24 (16.565), respectively. Cot, physical function (3/4, 6, and 12 months) = 74.32 (18.87); 73.51 (20.258); 80.97 (20.939), respectively
Moffet et al. [39], Canada	76 (Int = 38, Cot = 38)	Primary TKR	67.7	59.7	12 months	Int, pain (3/4, 6, and 12 months) = 9.6 (11.5); 8.9 (9.6); 9.4 (12.4), respectively physical function (3/4, 6, and 12 months) = 13.6 (15); 12.4 (14.4) 12 (14.8), respectively. Cot, pain (3/4, 6, and 12 months) = 17.2 (17.1), 16 (18.1), and 19.3 (17.5), respectively. Function (3/4, 6, and 12 months) = 18.9 (17.7), 18.6 (18.7), and 15.8 (17.6), respectively
Monticone et al. [40], Italy	110 (Int = 55, Cot = 55)	Primary TKR	67	64	12 months	Int, function (6 and 12 months) = − 82.58 (12.48), − 87.76 (8.95), respectively. Pain (6 and 12 months) = − 81.8 (13.26), − 87.35 (11.71), respectively. Cot, pain (6 and 12 months) = − 70.82 (19.58); − 77.38 (15.07), respectively; Function (6 and 12 months) =− 68.11 (19.23); − 75.78 (16.78)
Wang et al. [41], USA	520 (Int = 269, Cot = 251)	Patients who underwent Primary TKR	Int = 66.44 (8.10) Cot = 66.58 (8.19)	Int = 62.1 Cot = 57.8	12 months	Int, ROM (< 90°) = 0.35 (0.14) *p* = 0.47; SF-12 mental composite scores = 55.85 (7.68) *p* = 0.61 Cot, ROM (< 90°) = 0.32 (0.12); SF-12 mental composite scores = 56.15 (6.79)
Denis et al. [44], Canada	81 (Int -1 = 26; Int-2 = 28 Cot = 27)	Patients undergoing TKR	N/A	N/A	7 days	Int (E1): ROM flexion: 78.7 (10.6) *p* = 0.33; extension − 7.0 (3.7) *p* = 0.30; WOMAC score (pain) = 36.8 (15.6) *p* = 0.36 WOMAC (functional difficulty) = 40 (20.2) Int (E2): ROM flexion 83.3 (11.9); extension = − 6.3 (3.7); WOMAC (pain) = 27.7 (17.1); WOMAC functional difficulty (20.2) Cot, flexion = 80.4 (11.8); extension = − 8.0 (3.5); WOMAC (pain) = 39.8 (24.8); WOMAC (functional difficulty) = 33.0 (22.7)
Lenssen et al. [42], Netherlands	60 (Int = 30; Cot = 30)	Patients undergoing TKR	Int = 64.1 (8.1); Cot = 65 (9.1)	Int = 60; Cot = 70	6 weeks	Day 17 Int, active flexion ROM = 83.6 (11.4) *p* = 0.06; extension active = 6.3 (3.9) *p* = 0.11; WOMAC (pain) = 15.8 (4.7) *p* = 0.6; WOMAC (difficulty) = 49.1 (11.9) *p* = 0.23 Cot, active flexion ROM = 78.6 (8.7) *p* = 0.06; extension active = 8.1 (4.8) *p* = 0.11; WOMAC (pain) = 15.3 (4.1) *p* = 0.6; WOMAC (difficulty) = 45.3 (12.3) *p* = 0.23 6 weeks Int; active flexion ROM = 91.9 (13.6) *p* = 0.98; extension active = 6.3 (4.0) *p* = 0.63; WOMAC (pain) = 16.0 (3.7) *p* = 0.53; WOMAC (difficulty) = 53.0 (9.5) *p* = 0.92 Cot; active flexion ROM = 91.8 (14.3) *p* = 0.98; extension active = 6.9 (5.4) *p* = 0.63; WOMAC (pain) = 16.6 (4.0) *p* = 0.53; WOMAC (difficulty) = 52.7 (12.0) *p* = 0.92
Avramidis et al. [45], UK	30 (Int = 15; Cot = 15)	Patients undergoing TKR	Int = 68.20 (10.59); Cot = 71.20 (7.83)	Int = 66; Cot = 80	12 weeks	6 weeks, Int; walking distance = 176.1 m, Cot; walking distance = 151.7 m. MD = 24.4; *p* = 0.0002 12 weeks, Int; walking distance = 188.2 m Cot = 155.9 difference = 32.3 m *p* < 0.0001

*WOMAC * Western Ontario and McMaster Universities Arthritis Index, *KOOS* Change of Knee Injury and Osteoarthritis Outcome Score, *Int* intervention, *Cot* Control, *MD* Mean Difference, *GROC* Global Rating of Change, *ROM* Range of Motion, *OKS* Oxford Knee Score

**Table 4:** Description of studies on the cost-effectiveness of physiotherapy interventions for patient of TKR

Author, year, /location/study design/time-horizon	Target population	Intervention	Control	Outcomes/measurement used	Cost/perspective	Results (intervention vs control)	Quality assessment
Baulig et al. [46]/Germany/retrospective/3 weeks	# 8180 (median age varied between 72 to 75 years)	Inpatient rehabilitation	N/A	Staffelstein (pain, activity of daily living, and joint function) score increase of 0.24 (medians ranging from 0.17 to 0.34) between the departments	The median direct costs were estimated €2023 (departmental wise median €1679–€2283)/the health care insurer’ perspective	Cost-normalised effect estimates for the total cohort were in median 12% Staffelstein index increase per €1000 investment (department wise 9–16% per €1000)	22/24
Mitchell et al. [26]/UK/randomised controlled trial/12 weeks	#114 (mean age = 70)	Pre- and post-operative physiotherapy at home	Group exercises, usually once or twice a week	Health related quality of life (HRQoL) = 0.002 (− 0.034 to 0.039) WOMAC, functional daily living = − 1.0 (− 5.9 to 3.8); *p* = 0.677 pain = − 0.5 (− 2.0 to 1.0); *p* = 0.53 stiffness = − 0.2 (− 0.9 to 0.4), *p* = 0.496	The mean total costs of pre- and post-operative NHS services (Int = £5376; Cot = £5372) *physiotherapy services (Int = £197.9; Cot = £61.5) *transport costs (Int = 0, Cot = £38.7 per patient)	No ICER was reported	21/24
Kauppila et al. [47]/Finland/randomised controlled trial/12 months	#75 (mean age = 70.7 years)	A 10-day multidisciplinary rehabilitation programme	Standard physiotherapy	Intervention, WOMAC)- function = − 32.4 (26.4) pain = − 36.8 (25.8); control, WOMAC–Function = − 32.6 (20.1); pain = − 35.7 (20.4)	The mean direct total cost using the available cost data was €12,950 (median €12,018, IQR €10,293–15,759) in the multidisciplinary rehabilitation group (*n* 36) and €11,120 (median €9522, IQR €8524–11,091) in the control group (*n* 39). The mean direct total cost of rehabilitation services was €2005 (median €1792, IQR 1607–2224) in the multidisciplinary rehabilitation group and €490 (median €123, IQR €0–678) in the control group	Both protocols which were providing rehabilitation services turned out to be equally effective, but the conventional orthopaedic care protocol was unequivocally cost saving The saving was €1830 per patient (95% CI 548–3623) using the available direct cost data	23/24

*N/A* Not available

### Clinical effectiveness

Twenty-three of the included studies in this review reported the clinical effectiveness of physiotherapy interventions including range of motion (ROM), pain, functional performance, mobility, hospital length of stay, and health-related QoL (Table 3). The clinical outcomes reported in the studies included in the review are summarised below.

### Range of motion

**Fig. 2:**
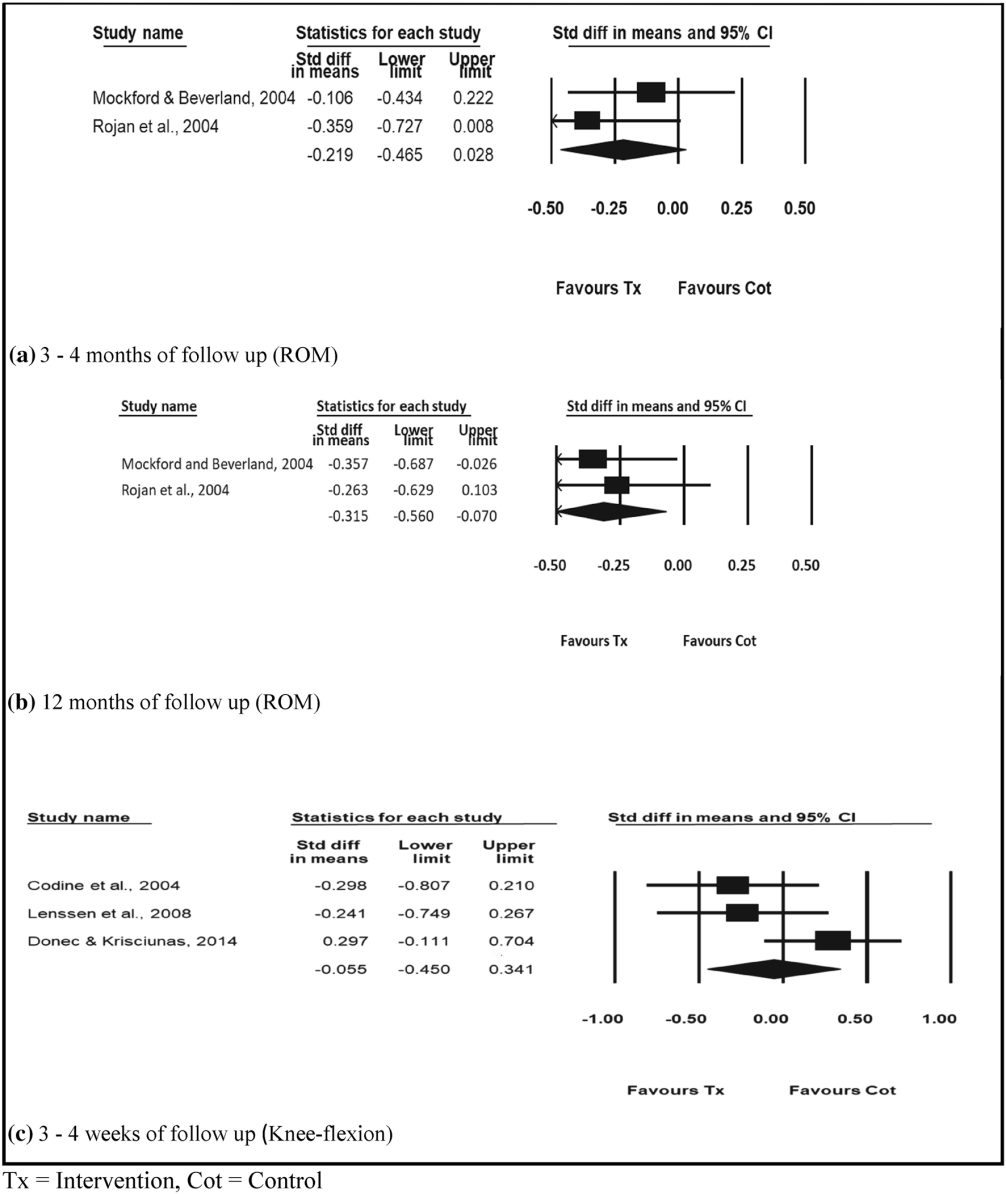
Physiotherapy exercise compared with control. **a** 3–4 months of follow-up (ROM). **b** 12 months of follow-up (ROM). **c** 3–4 weeks of follow-up (Knee-flexion). *Tx* Intervention, *Cot* Control

Seven studies included in the review reported ROM [32–36, 41, 42]. Random effect of meta-analysis for ROM in individuals with TKR at 3–4 months (Fig. 2a) and 12 months (Fig. 2b) showed that physiotherapy interventions were statistically significant compared to control with standard mean difference (SMD) − 0.219 [− 0.465 to 0.028], and − 0.315 [− 0.560 to 0.070], respectively.

Some of the included studies reported the clinical effectiveness of physiotherapy interventions on knee flexion ROM only and knee extension ROM only. Data on knee flexion ROM were available in five studies with 366 patients [23, 27, 28, 42, 43]. The meta-analysis of the three studies [23, 42, 43] indicated that patients that received physiotherapy interventions favoured the treatment group at 3 or 4 weeks, SMD − 0.055 [95% CI − 0.450 to 0.341] (Fig. 2c), however, this was not statistically significant.

**Fig. 3:**
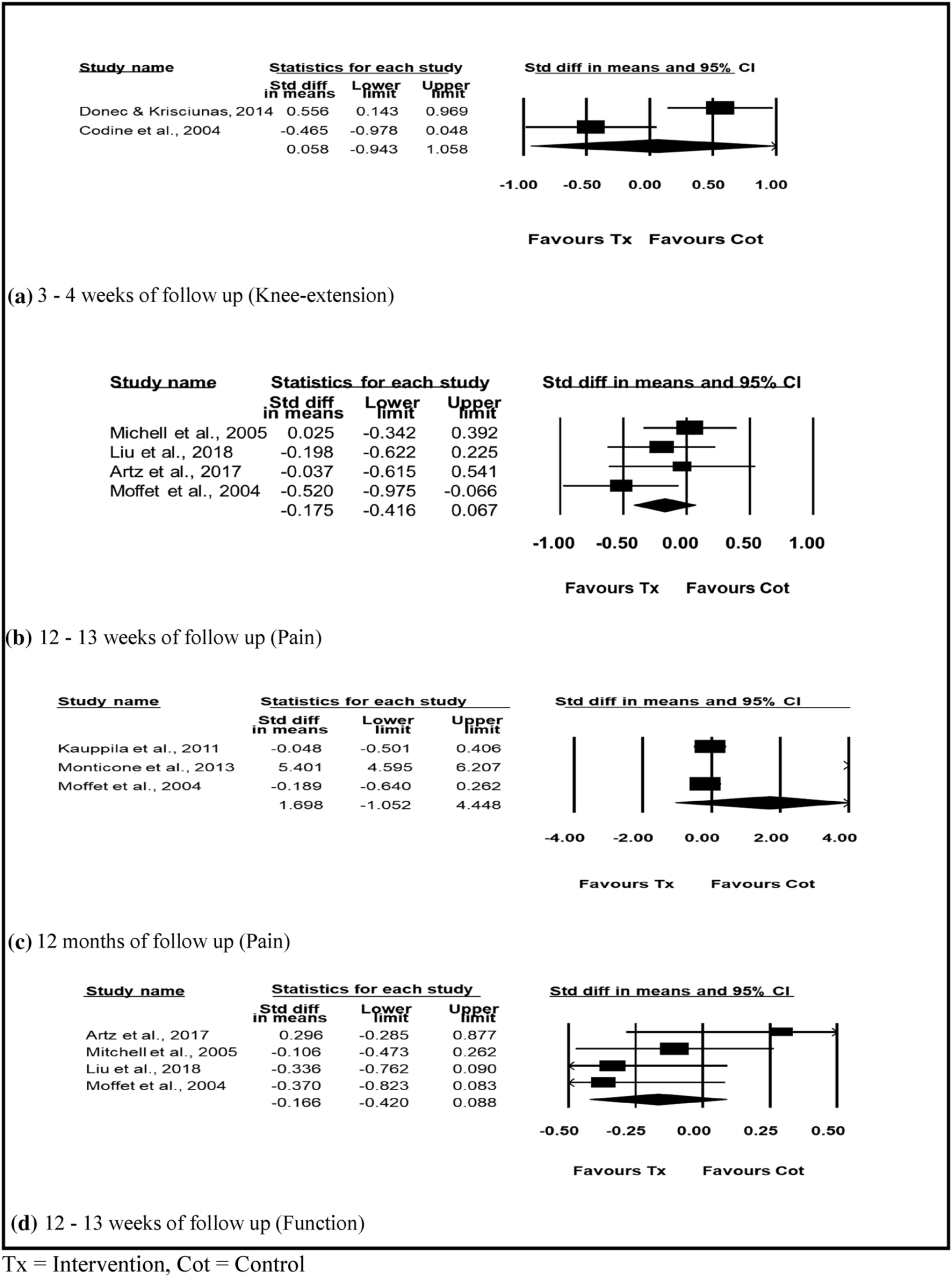
Physiotherapy exercise compared with control. **a** 3–4 weeks of follow-up (Knee-extension). **b** 12–13 weeks of follow-up (Pain). **c** 12 months of follow-up (Pain). **d** 12–13 weeks of follow-up (Function). *Tx* Intervention, *Cot* Control

Two of the included studies reported knee extension ROM on 150 patients [23, 43]. The individual studies reported that participants receiving physiotherapy interventions had improved knee extension ROM at the end of the rehabilitation. On the other hand, the random effect meta-analysis for knee extension comparing physiotherapy interventions with control showed no significant difference, SMD 0.058 [95% CI − 0.943 to 1.058] (Fig. 3a).

### Patient reported pain

Nine of the included studies reported the effect of physiotherapy interventions on knee pain scores [24, 26, 28, 29, 31, 32, 39, 40, 43]. The random effect meta-analysis for pain at 12–13 weeks showed that physiotherapy interventions favoured the treatment group, SMD − 0.175 [95% CI − 0.416 to 0.067] (Fig. 3b). Whereas, patients following TKR in the intervention group showed no benefit compared to control at 12 months (Fig. 3c).

### Function

Ten studies reported functional activity in patients following physiotherapy interventions [24, 26, 29, 31–33, 36, 37, 39, 40]. Random effect meta-analysis of four studies with 322 patients [24, 26, 31, 39] showed that functional activity of participants who received physiotherapy interventions improved their functional performance, SMD − 0.166 [− 0.420 to 0.088] (Fig. 3d).

### Mobility

Two studies reported the effect of electrical muscle simulation and acute weekend physiotherapy services on patient’s mobility [25, 45]. Compared to no or minimal intervention those participants receiving the physiotherapy interventions in both studies showed a statistically significant improvement in mobility I the short term [45] and in the long term [25].

### Cost-effectiveness

As indicated in Table 4, three studies on cost-effectiveness of physiotherapy interventions following TKR are included in this review [26, 46, 47]. Two studies [25, 46] were cost-effectiveness studies alongside randomised controlled trials, whereas the remaining one study was a retrospective cohort study [46]. All the studies included in this part of cost-effectiveness review considered direct costs from healthcare system and individual patient perspective. Two of the included studies reported that physiotherapy interventions for TKR were more expensive than the control and were not cost-effective [26, 47]. The intervention arm of one of the included studies contained a preoperative group exercise programme on the surgical ward, and guided subsequent exercise program at a 2 month outpatient control visit to an orthopaedic surgeon [26]. Likewise, the second [47] and third [46] study included in the review involved individual treatments such as preoperative visits and up to six post-discharge visits and rehabilitation, respectively.

## Discussion

To our knowledge, this is the first systematic review and meta-analysis that has examined the clinical and cost-effectiveness of physiotherapy interventions following TKR. The methodological quality of the included clinical effectiveness studies was assessed as adequate. Results of this meta-analysis of randomised controlled trials suggest that physiotherapy interventions improved the health status of the patients in terms of function, ROM, pain and mobility following TKR in a short term. With regards to the cost-effectiveness of physiotherapy interventions for patients following TKR, the findings of the current review indicate that it is unlikely to be cost saving from the health system perspective. The included cost-effectiveness studies demonstrated that home physiotherapy, and a multidisciplinary rehabilitation programme were clinically effective; however, they were resource intensive in terms of healthcare resources compared to the control.

Physiotherapy interventions improved knee flexion ROM in patients after undergoing TKR compared to a control. Patients in the intervention groups were provided with continuous passive motion for 2 consecutive hours twice daily [42], Kinesio Taping that helped them to achieve mechanical correction [43] and training sub maximally using eccentric isokinetic strengthening [23]. On the other hand, the random effect meta-analysis for knee extension ROM showed no statistical difference between the intervention and control groups [23, 43].

In relation to patient reported pain, we found two different findings depending on the duration of follow-up of patients. Patients in the intervention group showed a reduction in pain, whereas no statistical difference was found between the intervention and control group was shown at 12 months. Similarly, compared to the control the meta-analysis of randomised controlled trials showed that patients in the intervention group improved functional activity and ROM. The clinical effectiveness results from our meta-analysis is consistent with the previous reviews of physiotherapy exercise after TKR [14, 19]. These two reviews [14, 19] indicated that physiotherapy exercise resulted in improvements in physical function, ROM, and quality of life in short term. A previous review [14] has suggested that physiotherapy following TKR was not beneficial in the longer term.

The strengths and limitations of this review and meta-analysis should be considered. To provide reliable results, a rigorous appraisal of the evidence such as a prospective protocol, quality control of data and defined outcomes were considered. The present analysis included several studies involving approximately 30–520 patients, which directly affected the pooled estimate of the clinical effectiveness of physiotherapy interventions.

One of the limitations of the study was that the definition of physiotherapy in the literature remains very broad. As a result, the studies identified in this review contained different forms of physiotherapy interventions that might have affected positively or negatively to the evidence provided in this review. Second, results of trials with negative findings may not have been published. Third, although this study provides valuable information on the cost-effectiveness of physiotherapy interventions, aggregation of evidence is limited due to heterogeneity in terms of study design, economic perspective, outcomes measures and the cost categories included. Furthermore, this review only considered studies published with English language which may have limited its generalisability. Finally, due to the variation of outcomes measures and time of follow-up, it was not possible to combine studies on health-related QoL and mobility to reach meaningful conclusions. Overall, given that physiotherapy interventions are associated with improved knee flexion ROM, knee extension ROM, pain, function and ROM in a short term and ROM at 12 months. Thus, the findings suggest physiotherapy can be considered to be clinically effective for patients following TKR.

## Conclusions

The findings of this meta-analysis suggest that pain and function showed improvement in the short term with physiotherapy interventions following TKR. Moreover, the random effect of meta-analysis for ROM at 12 months showed that physiotherapy was beneficial compared to control. On the other hand, in the long-term patients reported no improvement in pain with physiotherapy interventions. Moreover, the results indicated that physiotherapy interventions for patients with TKR were neither cost-effective nor cost saving from health system perspective. Due to the nature of the evidence, particularly the uncertainty and small number of studies on the clinical effectiveness and cost-effectiveness, future studies should to properly monitor adherence to physiotherapy technique and provide high quality cost data. As evidence is continuing to emerge on the clinical and cost-effectiveness of physiotherapy, we recommend that our findings are periodically reviewed and revised.
